# Myocardial viability assessment and utility in contemporary management of ischemic cardiomyopathy

**DOI:** 10.1002/clc.23779

**Published:** 2022-01-25

**Authors:** Chandra K. Katikireddy, Arang Samim

**Affiliations:** ^1^ Department of Cardiology VA CCHCS UCSF Fresno California USA; ^2^ VA CCHCS UCSF Fresno California USA

**Keywords:** CMR, coronary revascularization, dobutamine stress echocardiograph, ischemic cardiomyopathy, myocardial viability, SPECT imaging

## Abstract

**Background:**

In clinical practice, we encounter ischemic cardiomyopathy (ICM) with underlying viable, dysfunctional myocardium on a regular basis. Evidence from the Surgical Treatment for Ischemic Heart failure (STICH) and its Extension Study is supportive of improved outcomes with coronary revascularization, irrespective of myocardial viable status. However, Dobutamine stress echocardiography (DSE) and single‐photon emission computed tomography (SPECT), used in STICH to assess myocardial viability may fail to distinguish hibernating myocardium from scar due to suboptimal image resolution and poor tissue characterization.

**Hypothesis:**

Cardiac magnetic resonance (CMR) and positron emission tomography (PET) can precisely quantify myocardial scar and identify metabolically active, viable myocardium respectively. Unlike DSE and SPECT, CMR and PET allow examining myocardial status as a contiguous spectrum from viable to partially viable myocardium with varying degrees of subendocardial scar and nonviable myocardium with predominantly transmural scar, the therapeutic and prognostic determinants of ICM.

**Methods:**

Under the guidance of CMR and PET imaging, myocardium can be distinguished viable from partially viable with subendocardial scar and predominantly transmural scar. In ICM, optimal medical therapy and coronary revascularization of viable/partially viable myocardium but not transmural scar may improve outcomes in patients with acceptable procedural risk.

**Results:**

Coronary revascularization of partially viable and viable myocardial territory may improve clinical outcomes by preventing future ischemic, infarct events and further worsening of left ventricular remodeling and function.

**Conclusions:**

When deciding if coronary revascularization is appropriate in a patient with ICM, it is essential to take a patient‐tailored, comprehensive approach incorporating myocardial viability, ischemia, and scar data with others such as procedural risk, and patient's comorbidities.

## Highlights

1. Viability of myocardium is assessed by different imaging modalities, probing different characteristics of the living myocyte – uptake of radioactive isotope, TC‐99m or Tl‐201 (SPECT MPI), contractile reserve (Dobutamine stress imaging, echo or CMR), metabolic properties (FDG uptake on PET), absence of scar (CMR).
2. Dysfunctional, viable myocardium as compared to nonviable myocardium carries a better prognosis with appropriate therapy.
3. Dysfunctional myocardium may not be simplified into a binary state of viable or not. It can be a continuum process, with a myocardial segment in a hybrid state with an intermix of viable myocytes in early to late phases of hibernation and fibrosis.
4. Nonviable myocardium on Dobutamine Stress Echocardiography or SPECT Nuclear imaging may be partially viable (with varying degrees of fibrosis) or viable with no scar, on CMR.
5. Coronary revascularization of partially viable or viable myocardium should be considered if the procedural risk is acceptable, as it improves long‐term outcomes by preventing further myocardial ischemia/infarction and possibly improving left ventricular function and remodeling.

## 1 INTRODUCTION

Left ventricular dysfunction and adverse remodeling in ischemic cardiomyopathy (ICM) from coronary artery disease denotes an adverse prognosis from recurrent myocardial infarction, heart failure, and arrhythmias. Ischemic ventricular dysfunction and remodeling with viable myocardium (VM) or partially viable myocardium (PVM) may have better outcomes when they receive appropriate therapy including coronary revascularization compared to those with predominantly fibrotic, nonviable myocardium (NVM).

**Figure 1:**
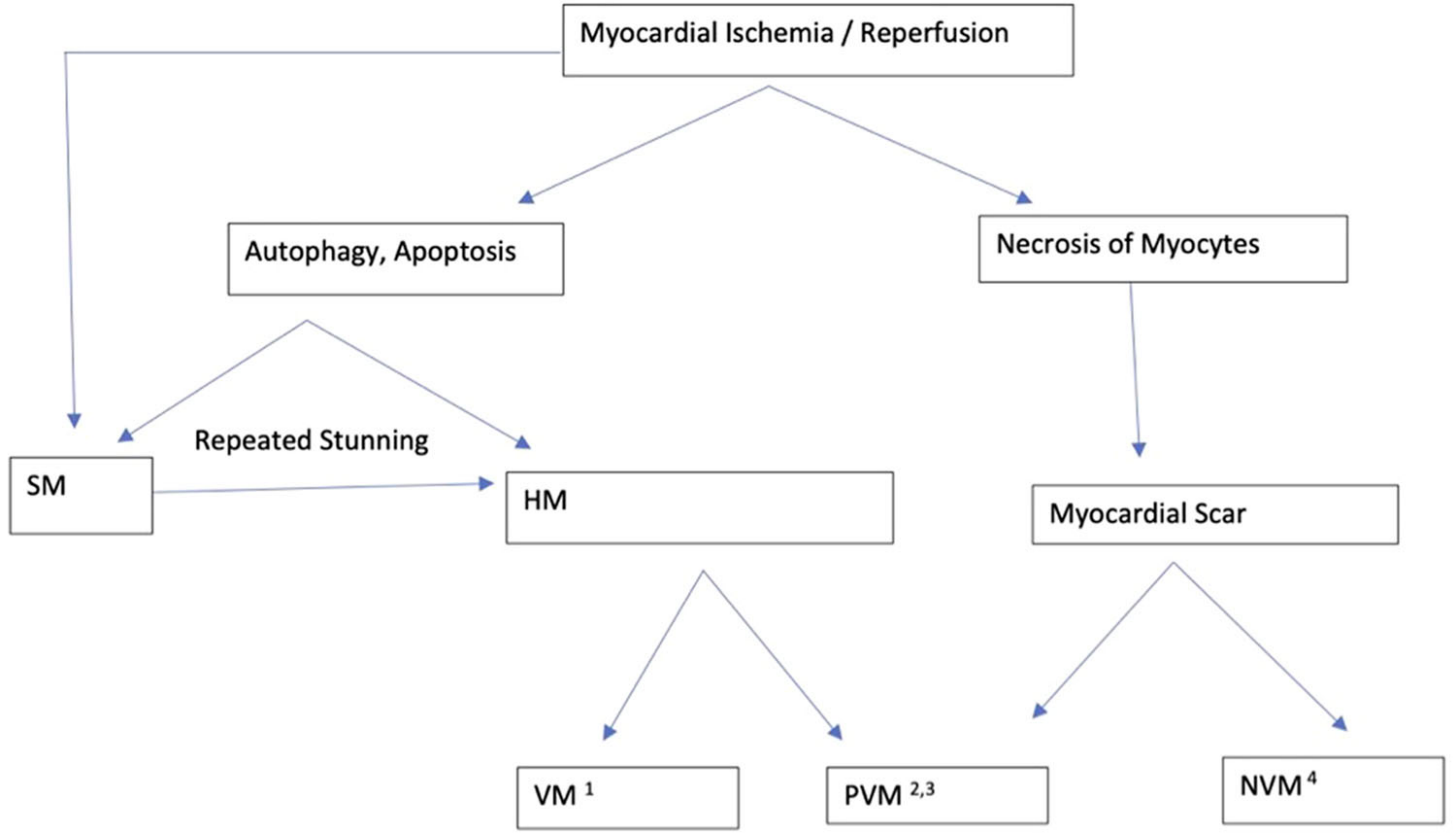
Consequences of myocardial ischemia/reperfusion. HM, hibernating myocardium; NVM^4^, nonviable myocardium with transmural scar; PVM^2^, partially viable, thinned myocardium in mid to late phases of HM; PVM^3^, partially viable myocardium with subendocardial scar; SM, stunned myocardium; VM^1^, viable myocardium in early to mid‐phase of HM

Myocardial ischemia leads to stunning, myocyte apoptosis, necrosis, inflammation, and fibrosis (Figure 1). Two types of myocardial dysfunction may happen in the setting of ischemia – stunning and hibernation.^1^, ^2^ Hibernating myocardium is a viable, dysfunctional state of the myocardium with a persistently reduced contractility due to reduced coronary blood flow at rest, which may be partially or completely reversible upon revascularization.^3^ Stunned myocardium is a dysfunctional state for a transient time from an episode of ischemia despite the restoration of normal blood flow.^1^, ^2^, ^3^ Chronic, repetitive stunning may lead to a hibernating myocardium in a short period of time.^4^ NVM, as compared to the two former viable states, is the result of irreversible necrosis of the myocytes leading to fibrosis and infarction.^1^, ^3^ It is important to recognize that progression of untreated ischemia and eventual replacement of hibernating myocardium by fibrosis without therapy is a chronic, continuum process.^3^, ^5^, ^6^ In a chronic state of ischemia, hibernating myocardium can be at early, intermediate, or late stages and prognostic outcomes depend on at which stage the therapeutic intervention is made (6). As such, hibernating myocardium may be VM, PVM, or near NVM intermixed with fibrosis.

The following sections of the paper discuss ‐ evaluation of myocardial viability by multimodality imaging, prognostic implications of VM, PVM, NVM; therapeutic decision making in ICM based on viability, left ventricular function, and remodeling.

## 2 MYOCARDIAL VIABILITY ASSESSMENT

In conventional clinical practice ‐ myocardium with contractile dysfunction is termed “VM” if it is predicted to recover contractility with medical therapy and coronary revascularization (CorR). However, with appropriate medical therapy and CoR, improved clinical outcomes may be noted despite the lack of a significant improvement in LV function.^7^ In light of this knowledge, we need to adopt a new approach to determine and use viability data in treating ICM.

**Figure 2:**
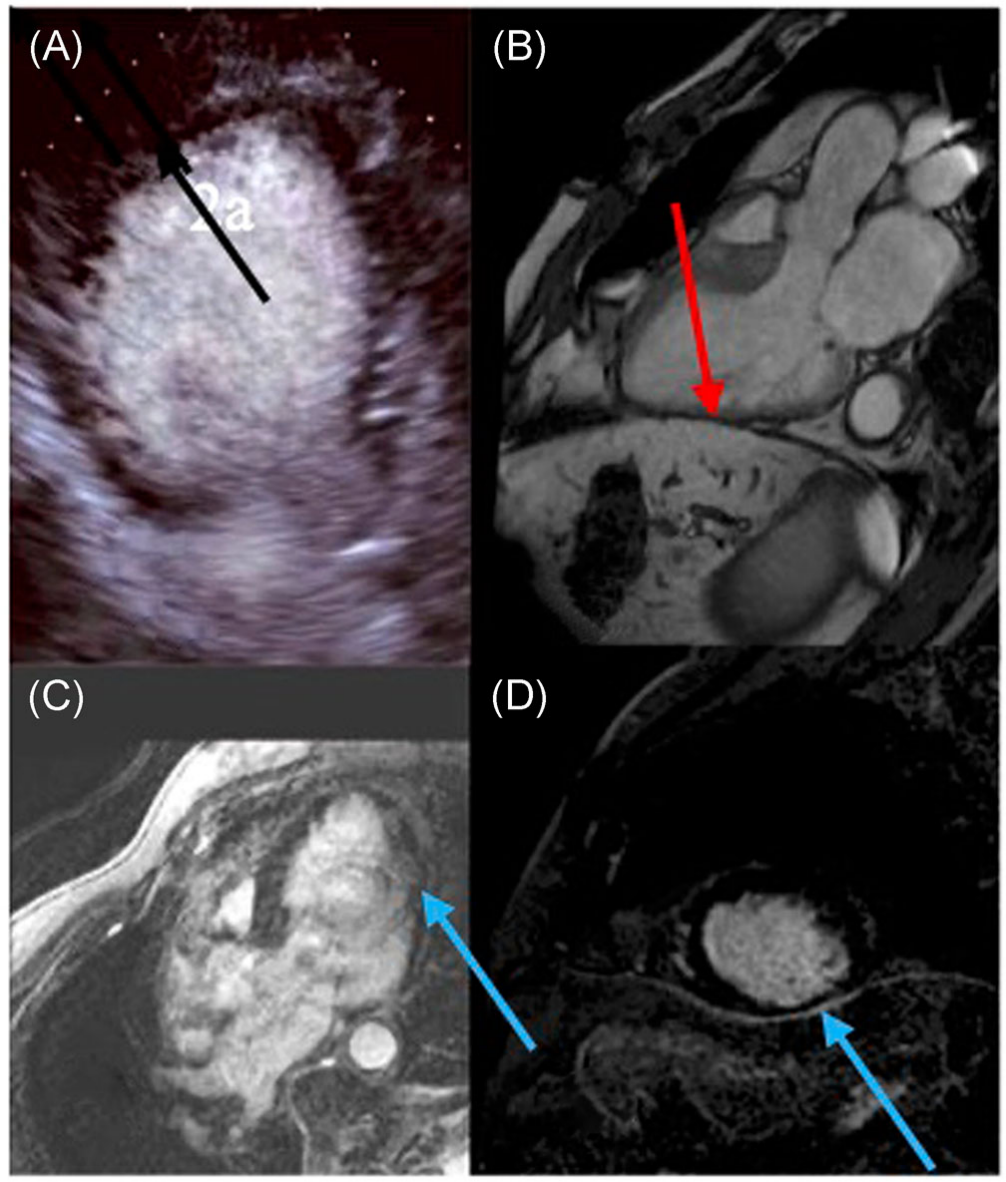
Inferolateral myocardial segment noted as NVM on Echo showed no scar (VM) on CMR. Inferolaterla wall on Echo (A) is thinned with no contractile reserve, suggestive of NVM. Thinned inferolateral wall on CMR (B) was shown to be VM with no LGE (C) and (D). (A) Black arrow: Thinned inferolateral wall on Echo, end diastole. (B) Red arrow: Thinned inferolateral wall on CMR, end diastole. (C) and (D): Blue arrow: Inferolateral wall on CMR with no LGE (scar). CMR, cardiac magnetic resonance; Echo, echocardiography; LGE, late gadolinium enhancement; NVM, nonviable myocardium; VM, viable myocardium

**Figure 3:**
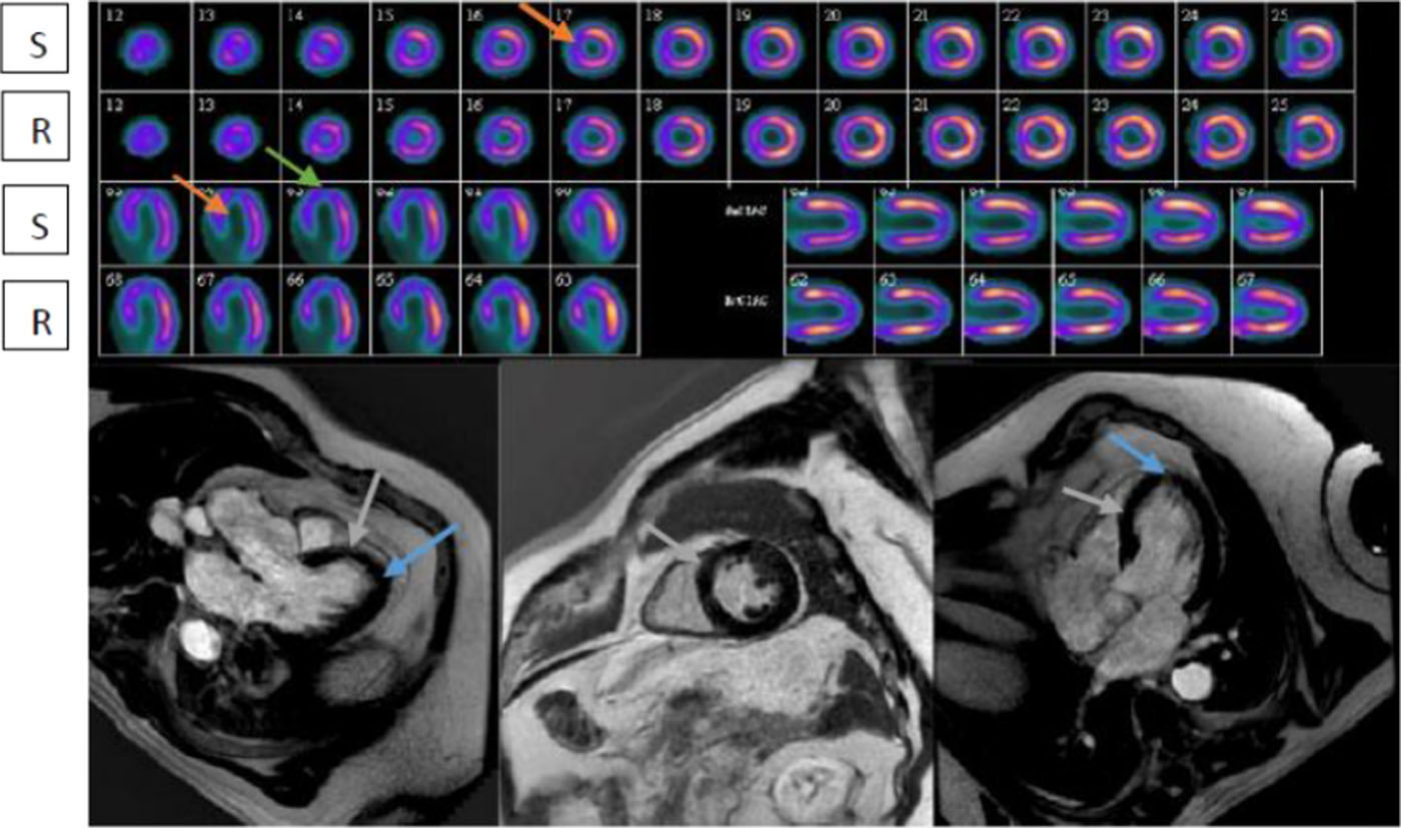
Fixed perfusion defect of the apex and septum on SPECT suggests infarct, however, characterized as VM on CMR with no LGE. LV septum (red arrow) and apex (green arrow) reveal fixed perfusion defect on strss and rest SPECT imaging (A) suggestive of NVM; on CMR (B) septum (gray arrow) and apex (blue arrow) reveal VM (no scar on LGE imaging). CMR, cardiac magentic resonance; LGE, late gadolinium enhancement; NVM, nonviable myocardium; R, rest attenuation corrected; S, stress attenuation corrected; SPECT, Single Photon Emission Computed Tomography; VM, viable myocardium

Current myocardial viability imaging techniques interrogate structural and functional integrity of the cardiac myocyte and its intracellular processes; end‐diastolic wall thickness on echocardiography (Echo) or cardiac magnetic resonance (CMR), contractile reserve assessment with low dose dobutamine by Echo or CMR, cell membrane integrity based on radioactive tracer uptake on single‐photon emission computed tomography (SPECT), ischemic myocardial metabolic properties (cellular glucose uptake) by positron emission tomography (PET), and late gadolinium hyper‐enhancement and T1 mapping (myocardial fibrosis/replacement scar) by CMR. When VM is defined in a traditional way as the myocardium with improved functional recovery post revascularization, tests (dobutamine Echo and CMR) that evaluate contractile reserve possess higher positive predictive value and specificity but lower sensitivity in comparison to those that estimate cell membrane integrity (SPECT) and metabolic (PET) properties, which are known to have higher negative predictive value and sensitivity but lower specificity.^3^, ^5^, ^8^ Of note, predominantly, the data comparing the diagnostic ability of different imaging tests were not derived from the same studies or subjects. Diagnostic accuracy of different imaging modalities in estimating the viable status of the myocardium is comparable. However, distinguishing PVM with subendocardial scar or thinned, hibernating myocardium from NVM may not always be feasible on Echo or SPECT, a noteworthy limitation of these techniques (Figures 2 and 3).

Useful modalities in viability assessment in the clinical practice are discussed below (Table S1).

## 3 ELECTROCARDIOGRAM (ECG)

The ECG is an initial tool in the evaluation of viability. While the absence of pathologic Q‐waves may be suggestive of viable myocardium^9^ and the presence of them may imply infarct, Q waves are not specific for myocardial infarct and are seen in myocardial hypertrophy, WPW, and rarely hibernating myocardium.^10^ The presence or absence of Q waves information can be a helpful complementary marker in conjunction with the other imaging parameters and clinical data to determine myocardial viability.

## 4 2D ECHOCARDIOGRAPHY

Normal segmental thickness (>6 mm) hints of VM and severely thinned (4 mm or less) segments may be suggestive of NVM or PVM. Normal end‐diastolic wall thickness was shown to have a high sensitivity (>90%) and low specificity (<50%) in a meta‐analysis in predicting LV contractile recovery.^11^

## 5 DOBUTAMINE STRESS ECHOCARDIOGRAPHY (DSE)

Stress echocardiography using low dose Dobutamine to elicit contractile response in viable segments with systolic dysfunction, is a low risk, quick, noninvasive test with no radiation risk. VM with a contractile reserve in a minimum of >5 segments, increases the success rate of functional recovery following coronary revascularization.^12^

### 5.1 DSE viability testing protocol

Typically, to induce a contractile response in baseline dysfunctional segments, Dobutamine is started at low doses (2.5–5 μg/kg/min) and gradually increased to intermediate doses (up to 20 μg/kg/min). Subsequently, to assess ischemic response, infusion is incrementally increased to stress doses (up to 40 μg/kg/min), with the addition of IV Atropine as needed (up to 2 mg in divided doses), to achieve a target heart rate (85% (220‐age). Under close hemodynamic and EKG monitoring, Dobutamine is infused for 3 min at each stage but infusion time can be variable based on patient's hemodynamics and contractile response.^13^

Four distinct responses to dobutamine stress have been described. A biphasic response, in which contractility improves with low dose dobutamine and worsens at higher doses due to ischemia has been shown to be 60% sensitive and 88% specific in predicting contractile recovery 6 weeks after coronary revascularization.^14^ Second, PVM with reduced perfusion reserve may have no contractile reserve and DSE may further worsen contractility. Third, when there is a sustained improvement in contractility with increasing dobutamine dose, it is believed to be the myocardium with the restored, normal flow, nontransmural infarction, or remodeled. Finally, if the myocardium is scarred and NV, no contractile response to dobutamine is expected to be seen,^15^ however this finding lacks high sensitivity to detect NVM (Figure 2).

DSE is widely available, accessible and inexpensive with no radiation risk. If the positive contractile reserve is demonstrated on DSE, it is a specific sign of VM. However, the absence of contractile reserve on DSE may not preclude viability in advanced stages of thinned, hibernating myocardium. Limitations of DSE include poor acoustic windows and other technical difficulties in image acquisition that may result in images of suboptimal, non‐diagnostic quality.

## 6 SINGLE‐PHOTON EMISSION COMPUTERIZED TOMOGRAPHY (SPECT)

In SPECT imaging, VM is illustrated from detecting the positive uptake of the radionuclide‐labeled tracer by a myocyte with an intact cell membrane. An arbitrary cutoff > 50% tracer activity (normal to mildly reduced counts) of the maximum uptake of a normal segment, has been used to identify VM, 30%–50% suggests PVM (moderately reduced counts) and < 30% indicative of NVM (severely reduced to absent counts). When rest perfusion defect is suggestive of equivocal viability, stress perfusion imaging may be helpful, as a worsening defect on stress (ischemic response) would be indicative of PVM/VM. In addition, relatively intact wall motion and thickening at rest or improved segmental function on poststress gated imaging would be a strong marker of the absence of transmural myocardial scar.

The most common tracers used in SPECT myocardial perfusion imaging are ^201^Tl (Thallium) and ^99m^Tc. ASNC 2018 SPECT imaging guidelines provide a detailed description of rest/stress perfusion imaging of ^99m^Tc and stress/rest, 4/24 h delayed redistribution imaging of ^201^ Tl with or without reinjection.^16^ A brief summary of salient points of SPECT viability imaging is noted below.

### 6.1 ^201^ Tl viability protocol

The initial myocardial uptake of ^201^ Tl is driven by blood flow state at rest whereas the subsequent uptake over next 4–24 h is determined by “refill and redistribution” of the isotope, determined by cellular membrane integrity.^5^, ^8^ Hibernating myocardium may appear as a perfusion defect on early images due to impaired blood flow at rest but normalizes on 4–24 h delayed imaging (with or without reinjection of ^201^Tl) from redistribution of the ^201^Tl. Sensitivity of viability detection on ^201^Tl imaging may increase in late (24 h) reinjection/redistribution protocol compared to 4 h early redistribution protocol. Radiopharmaceutical activity of ^201^T (t ½ 73 h) in rest/redistribution imaging is approximately 3mCI with a corresponding radiation effective dose of 10–15 mSV.

### 6.2 Tc^99m^ viability protocol

Tc^99m^ myocardial uptake is dependent on a passive cell membrane diffusion and mitochondrial retention with no redistribution property. Rest and stress perfusion imaging are typically performed the same day, however, a 2‐day protocol may be used when higher tracer doses are required for both rest and stress, to overcome a suboptimal image quality from soft tissue attenuation (from a large body habitus). Radiopharmaceutical activity of Tc^99m^ (t ½ 6 h) for rest imaging is approximately 10 mCI (2–3 mSv), typically a 1/3 of stress imaging, in same day rest/stress protocol. After injecting Tc^99m^, approximately 30–60 min wait time is allowed before imaging the heart, to increase the clearance of subdiaphragmatic tracer activity and reduce the incidence of artifacts as a result of. Soft tissue attenuation and motion artifacts may appear as perfusion defects that can be overcome by using attenuation and motion correction software. Imaging in prone positioning may help in eliminating a diaphragmatic attenuation artifact.

When an artifact is ruled out, a perfusion defect at rest on Tc imaging is either an infarct (transmural or subendocardial) or hibernating myocardium (Figure 3). A further distinction of these fixed perfusion defects may be determined by the presence of wall motion and thickening on gated imaging (rest or poststress) and worsening of perfusion defect with stress in hibernating myocardium. The advantage of ^99m^Tc‐sestamibi is its much shorter protocol. Even though Tl is considered superior to Tc imaging due to its redistribution feature, studies have shown that both are comparable in diagnostic accuracy in detecting VM.^4^, ^5^ The sensitivity and specificity of ^201^Tl were demonstrated to be 86% and 59%, respectively, for predicting functional recovery after revascularization and 81% and 66% for Tc^99m^, respectively.^5^, ^8^ Nitrate administration may further enhance the sensitivity of SPECT to detect VM.^17^

SPECT imaging is easily available and lower cost as compared to PET imaging. While normal uptake of radioactive tracer confirms the viability of myocardium, absence of tracer uptake does not confirm nonviable, transmural myocardial scar, as discussed below.

## 7 NVM ON SPECT AND ECHOCARDIOGRAPHY

**Figure 4:**
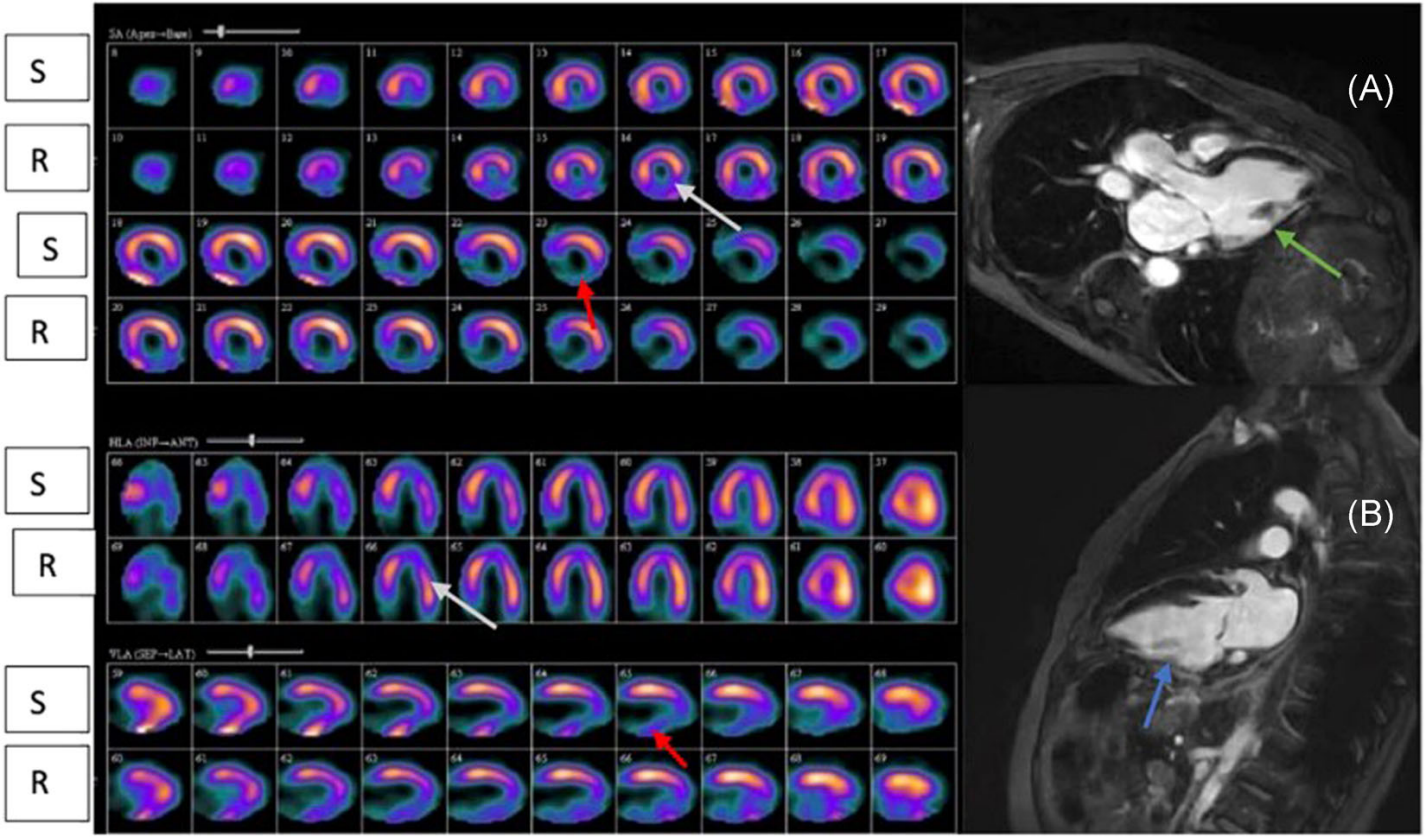
(A) PVM on SPECT showed subendocardial infarct on CMR. (B) NVM on SPECT confirmed as a transmural scar on CMR. (A) Inferolateral wall on SPECT that is partially reversible on rest imaging (gray arrow), suggestive of PVM; inferolateral wall on LGE of CMR reveal subendocardial infarct of <50% transmural extent (green arrow). (B) Inferior wall perfusion fixed defect on stress and rest SPECT imaging (red arrow), indicative of NVM and confirmed by CMR as transmural scar (blue arrow). CMR, cardiac magentic resonance; LGE, late gadolinium enhancement; NVM, nonviable myocardium; PVM, partially viable myocardium; R, rest attenuation corrected; S, stress attenuation corrected; SPECT, Single Photon Emission Computed Tomography; VM, viable myocardium

NVM by SPECT and Echo imaging techniques has been conventionally assumed as myocardial scar in totality. Lack of superior resolution to detect radioactive isotope uptake by thinned viable myocardium on SPECT and absence of contractile reserve in thinned non‐fibrotic myocardial segments on Echo may result in labeling of VM or PVM as NVM. Myocytes of the early hibernating phase coexisting with those in late stages may appear as a perfusion defect with <50% radioactive isotope activity of a normal segment. Differential characterization of NVM on SPECT and Echo would be (1) thinned, advanced stages of hibernating myocardium (Figure 3), (2) a mix of subendocardial infarct and viable epicardial myocardium (Figure 4A), and (3) predominantly scar (Figure 4B).

## 8 POSITRON EMISSION TOMOGRAPHY (PET)

PET imaging uses the preserved metabolic property of viable myocardium as opposed to the absence of metabolic activity in the scar. Superior resolution, quick imaging, absolute quantification of myocardial perfusion, and less radiation exposure are a few advantages of PET over the SPECT.

Cardiac PET uses N‐13 ammonia or Rubidium‐82 (^82^Rb) to assess perfusion and F18‐Fludeoxyglucose (^18^F‐FDG) to evaluate myocardial glucose metabolism.^5^, ^8^ At rest, healthy myocardium oxidizes free fatty acids to produce ATP. In the setting of myocardial ischemia, there would be a shift of hibernating myocardial metabolism from fatty acids to glucose with upregulation of glucose transporters.

For optimal ^18^F‐FDG uptake of viable myocardium, it is crucial to stimulate endogenous insulin release by appropriate dietary protocol, oral or IV glucose loading, and if needed insulin supplementation, to achieve appropriate serum glucose (100–140 mg/dl) levels before injecting ^18^F‐FDG. Suboptimal patient preparation may yield poor, non‐diagnostic images. Preparation of diabetic patients can be particularly challenging, requiring insulin injection to overcome myocardial insulin resistance and may take longer wait times from injecting ^18^F‐FDG to image acquisition. PET imaging is performed about 45–90 min (up to 3 h in diabetics) after injecting approximately 10 mCi (7 mSv) of ^18^F‐FDG (t ½ 110 min). For the purpose of attenuation correction, low resolution, nongated CT transmission scanning is performed afterwards. ASNC 2016 guidelines provide a detailed description of the patient preparation and PET imaging protocols.^18^

Viability testing by PET consists of rest imaging with perfusion tracer and metabolic imaging with ^18^F‐FDG. Normal rest perfusion imaging confirms viability by demonstrating intact myocyte cell membrane, eliminating the need for metabolic imaging. VM is characterized by reduced resting perfusion and preserved ^18^F‐FDG uptake.^19^ Meta‐analyses have indicated a superior diagnostic accuracy of PET in comparison to other modalities to detect VM.^5^ Limitations of PET include the lack of readily available scanners due to high cost, the complex, lengthy patient preparation protocols. Other shortcomings of PET imaging include, its inability to detect small subendocardial infarcts and variability of FDG uptake which can be impacted by certain medications (levothyroxine), diabetes, age, sex, and heart failure (20).

## 9 CARDIAC MAGNETIC RESONANCE IMAGING (CMR)

Ability to directly image and estimate the scar burden, myocardial perfusion, segmental wall motion, thickness, and contractile reserve with dobutamine infusion, left ventricular ejection fraction, and ventricular volumes,^5^, ^15^ makes CMR an ideal test to evaluate VM in a comprehensive manner.

### 9.1 CMR viability assessment protocol

#### 9.1.1 Late gadolinium enhancement (LGE) imaging

Gadolinium contrast is rapidly cleared from normal myocardium within 10 min of injection. However, in case of myocardial scar, the contrast is trapped with a delayed clearance from the increased interstitial space. In LGE CMR sequence, while the normal myocardium with no contrast retention is nulled (dark appearance), myocardial scar appears bright from retained contrast.^19^ Due to superior spatial resolution, CMR can accurately quantify the extent and transmurality of the myocardial scar (LGE) and viable myocardium.^5^ If transmurality of LGE of a myocardial segment is greater than 50%, it is considered NVM, as a measure of lack of contractile recovery following CorR.^5^

#### 9.1.2 Low dose dobutamine stress MRI

If LGE is <50% of segmental thickness or if myocardium is severely thinned and akinetic, the predictive accuracy of functional recovery with therapy can be further enhanced by demonstrating contractile reserve with low dose dobutamine.^5^

Updated version of CMR standardized protocols from 2020 provide, further details of LGE imaging.^20^ Of note, CMR contraindications such as claustrophobia, non‐ MRI conditional implanted devices and selective metallic implants preclude the utility of CMR. Recent development of MRI conditional pacemakers/ICDs and advancements in imaging technique to reduce the device artifact (wideband LGE sequence), allow CMR feasible in some patients with these devices. In advanced renal disease with GFR < 30 ml/m^2^, gadolinium‐based contrast media (GBCM) may be relatively contraindicated due to the risk of nephrogenic fibrosing sclerosis (NFS). However, a joint consensus statement from the American college of Radiology and the National kidney foundation advocates a more liberal use of Group II and Group III GBCM, by weighing potential benefits of GBCM based MRI study versus extremely low risk of NSF.^21^ In non‐contrast MRI, abnormally elevated T1 values on native T1 mapping sequence, may be suggestive of myocardial fibrosis in appropriate clinical context, however, this nonspecific marker is not diagnostic by itself. Integration of multiple viability markers; end‐diastolic segmental thickness, LGE, and contractile reserve with dobutamine would yield high sensitivity and specificity for determination of variable degrees of myocardial viability ‐ VM (<50% LGE with contractile reserve), PVM (non‐transmural LGE or very thinned myocardium with absence of contractile reserve) and NVM (transmural scar).^5^, ^8^, ^22^

In the STICHES trial (7), no significant contractile recovery of dysfunctional myocardium was demonstrated in the group (surgical CoR arm) despite improved outcomes – a finding that suggests any residual myocardium in the absence of transmural scar may benefit from CorR.

### 9.2 Other techniques/modalities

Other imaging techniques and markers ‐ Myocardial contrast echocardiography (microvascular integrity assessment), speckle echocardiography (myocardial strain and strain rate imaging), and Cardiac CT (myocardial perfusion and scar imaging by late hyper‐enhancement), have been studied to assess myocardial viability.^23^, ^24^, ^25^ At present, none of these are used in mainstream clinical practice due to lack of familiarity, unavailability with no clear evidence of improved diagnostic accuracy over the commonly used methods as detailed above.

## 10 CHOOSING THE VIABILITY TEST

Viability testing appears to be most helpful when it is uncertain that the myocardial segment (s) in question is predominantly transmural scar or otherwise, due to the clinical implications of better prognosis of dysfunctional PVM and VM by medical therapy and CorR.^7^, ^26^, ^27^, ^28^, ^29^, ^30^

If the dysfunctional myocardial segment possesses relatively preserved thickness with wall motion no worse than hypokinesis and absence of Q waves on EKG, it is unlikely that segment is NVM (scar), precluding need for any further testing to assess viability.

Viability testing should be tailored to the individual patient based on several factors including limitations or contraindications of a particular study in each patient, local expertise, and availability.

The degree of LV remodeling and dysfunction may play a role in deciding which test to perform. Patients with extreme degrees of LV dilatation and segmental wall thinning may need an advanced imaging modality (CMR, PET) that could distinguish PVM from NVM (scar). In patients with mild to moderate degree of LV dysfunction and remodeling, dobutamine stress Echo and SPECT imaging may suffice.

## 11 MYOCARDIA VIABILITY STATUS – THERAPEUTIC AND PROGNOSTIC IMPLICATIONS

Ischemic cardiomyopathy subjects with VM appear to have a better prognosis with medical therapy and CorR compared with those with large amounts of scar burden who are at higher risk for heart failure and ventricular arrhythmias.^27^, ^28^ Many observational studies and meta‐analyses have demonstrated that revascularization of dysfunctional, ischemic, VM but not NVM results in improved left ventricular function leading to better clinical outcomes.^26^ These studies were from the era before making significant advancements in medical therapy of ischemic cardiomyopathy.

The Surgical Treatment for Ischemic Heart Failure (STICH), a prospective, randomized study examined 5‐year mortality outcomes in severe, ischemic LV dysfunction (EF < 35%) in the surgical revascularization and medical therapy arm as compared with medical therapy alone group and found no difference. A viability sub‐study found no interaction between the viability status of myocardium and the clinical outcomes. Subsequently, the STICH Extension Study (STICHES), a 10‐year long‐term follow‐up study found improved mortality outcomes in surgical revascularization group; however, yet again the viability sub‐study of STICHES showed no association of myocardial viability status with the outcomes. In addition, improved outcomes were noted to be independent of improvement in LV systolic function.

How to reconcile the above findings has been an intense debate as it goes against the logic that dysfunctional myocardium with diminished perfusion when rescued with CoR, should translate into improved outcomes ‐ an established notion supported by wealth of prior observational data. A close examination of STICH study patient population, limitations of methods and imaging techniques used for viability, and further follow‐up LVEF assessment may provide a few clues.

To begin with, the findings may not be reproducible with generalized applicability as the viability sub‐study of STICH was a non‐randomized, non‐blinded, underpowered study with a small number of subjects with NVM. In this patient population with a severely dilated and dysfunctional LV with thinned and severely hypokinetic to akinetic segments, simply classifying myocardium as viable or nonviable in a binary fashion with no appreciation of varying degrees of PVM that may influence the outcomes of therapy might have led to these findings that can't be explained in a clinically intuitive sense. Moreover, SPECT or dobutamine echocardiography imaging techniques used in STICH study lack the ability to directly image myocardial scar and characterize and distinguish the thinned myocardium as hibernating versus subendocardial or transmural scar, as each portends a different prognostic significance.

The LVEF improvement with therapy that was claimed to be not associated with improved outcomes in STICHES trial was minimal (2%), that may be statistically but not clinically significant. In addition, a single follow‐up LVEF measurement was performed a bit prematurely at 3 months, as therapy may take a considerably longer time (>3–6 months) to result in a meaningful improvement in EF (>5%) in a severely dilated, dysfunctional LV.^31^

Plausible underlying mechanisms for the improved outcomes noted in surgical revascularization group in STICHES are: 1. CoR may prevent acute MI and complications, salvaging any further damage of VM and PVM (31). Trials comparing CABG versus PCI and the ISCHEMIA trial support the fact that CorR reduces future spontaneous myocardial infarction (MI) (32–34). 2. Improvement in LV systolic function (EF) from CoR over the long‐term (35). 3. Revascularization may not improve resting LVEF when baseline LV remodeling and systolic function is severely reduced, however, CoR may aid in preserving myocardial contractile reserve with stress/exercise. 4. CoR may reduce the excitability of arrhythmogenic foci and incidence of ventricular arrhythmias.

The deterministic nature of the myocardial viability status on clinical outcomes of revascularization in ischemic cardiomyopathy has been observed in a few prospective studies.^31^, ^32^

## 12 THERAPEUTIC DECISION MAKING IN CLINICAL PRACTICE

**Figure 5:**
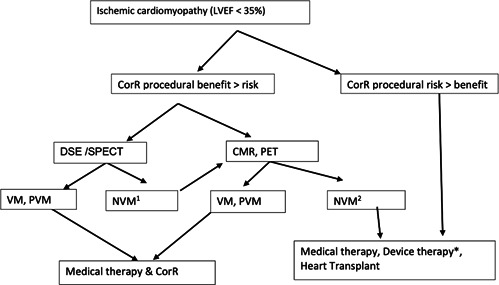
Viability imaging and management approach. CMR, cardiac magentic resonance; CorR, coronary revascularization; Device therapy*: cardiac resynchronization therapy (CRT), left ventricular assist device and mitral/tricuspid clip valve repair; DSE, dobutamine stress echo; LVEF, left ventricular ejection fraction; NVM1, nonviable myocardium on DSE/SPECT, NVM2, nonviable myocardium on CMR (transmural scar); PET, positron emission tomography; PVM, partially viable myocardium; SPECT, Single Photon Emission Computed Tomography; VM, viable myocardium

Due to the conflicting and controversial data as noted above, there has been an ongoing debate regarding viability testing and CorR in patients with ischemic left ventricular dysfunction (Figure 5).

We must keep in mind that many other factors such as clinical symptomatology, procedural risk, anatomical extent and complexity of CAD, degree of LV remodeling – should be considered besides myocardial viability and left ventricular scar burden information in making the CorR decision.

### 12.1 VM

When dysfunctional myocardium is determined as viable by any testing modality, prognostic outcomes improve with CorR of anatomically corresponding coronary territory. This is applicable in both mild to moderate (EF > 35%) and severe (EF < 35%) LV dysfunction and remodeling.^26^, ^27^, ^28^ Evidence from prospective, randomized clinical trials is sparse when it comes to surgical versus percutaneous CoR choice in LV dysfunction. However, CABG is preferred over PCI in patients with multivessel CAD, diabetes, and complex coronary anatomy due to improved survival and reduced cardiac events with surgery.^33^, ^34^ Lack of mortality benefit with percutaneous intervention may be partly due to exclusion of high‐risk patients such as LM disease, utilization of the older generation stents, and subpar medical therapy and unlike CABG, PCI would not fix the non‐obstructive lesions, a potential source of future MI.

In ischemic cardiomyopathy with advanced LV systolic dysfunction, it is essential to optimize medical therapy and take a multitude of factors; presence or absence of angina, degree of myocardial ischemia, the extent of scar burden in the territory of anatomical CAD, LV adverse remodeling, LVEF, patient's comorbidities, and procedural risk into consideration before coronary revascularization.

In general, left ventricular hibernating myocardium of approximately 20% of LV mass may be needed to make a meaningful impact in LV function improvement (at least >5% LVEF) after CorR.^35^ On the same token, when the myocardial scar is >20% of LV myocardium or the number of scar segments >4, the success of LV global functional recovery with CR is less likely.^33^ LV function improvement following the revascularization therapy may take from 6 months to a year or even longer in severely dysfunctional cases.^34^

### 12.2 NVM on SPECT and Echo

When myocardium is noted as NVM on SPECT nuclear or echocardiography but there is a suspicion of PVM based on clinical, EKG and other imaging parameters, it would be reasonable to use CMR to confirm if the NVM is predominantly scar versus PVM. After optimizing medical therapy, If the patient's surgical risk is acceptable, CorR of the myocardial segments with PVM with no transmural scar should be considered.^7^, ^35^ In addition, CMR would provide an accurate assessment of the degree of LV remodeling (volumes and ejection fraction), the prognostic data that can be used pre ‐ and postrevascularization (5).

#### 12.2.1 NVM on SPECT and echo with no CMR, PET availability

Myocardium may be noted as NVM on SPECT or Echo, however, at times, no advanced imaging (CMR, PET) is feasible due to either contraindication, lack of availability, or affordability. As discussed earlier, the possibility of labeling VM or PVM as NVM on Echo and SPECT is not uncommon. In addition, as mentioned above, ICM patients from STICHES study who underwent surgical CorR benefited regardless of viability status on SPECT or Echo. In these circumstances, it would be prudent to make the decision by a multidisciplinary team of a general cardiologist, an interventional cardiologist, a cardiac surgeon, an advanced cardiac imager, and a heart failure expert to discuss each patient on a case‐to‐case basis. In addition to myocardial viability status, patients should be evaluated for the degree of myocardial ischemia, LV adverse remodeling, LV dyssynchrony, and associated functional mitral and tricuspid insufficiency. In severe LV dysfunction (EF < 35%), patients should also be assessed for the feasibility and eligibility for other advanced therapeutic options such as cardiac resynchronization therapy, Mitral/Tricuspid valve intervention for functional insufficiency, LV assist device where appropriate, with or without CorR. When considering for CorR, it is most important to take the patient's functional status, symptomatology, and comorbidities into account and assess the overall procedural risks versus benefits of CorR. For instance, CorR may be appropriate in a patient with chest pain symptoms, evidence of myocardial ischemia, PVM/VM on SPECT/Echo with low to intermediate procedural risk; however, the risk of CoR may counterbalance or even outweigh the benefits, in a patient with no ischemic symptoms, advanced CHF with a severely remodeled LV and notable surrogate markers of myocardial scar such as akinetic, thinned myocardium on Echo, absent radioactive tracer uptake on SPECT and q waves on EKG.

### 12.3 NVM on CMR and PET imaging

Revascularization of predominantly scarred or metabolically inactive segments may not be warranted as it is not expected to improve the outcomes.^32^, ^36^

## 13 CONCLUSIONS

Dysfunctional myocardium should not be distinguished as a binary state, viable or nonviable as it is rather a continuum, ranging from early stages of hibernation with minimal LV remodeling and no fibrosis to an intermediate phase of thinned, partially viable, and partially fibrotic state and finally a terminal, primarily a fibrotic phase.^6^ Viable or partially viable myocardial segments should be considered for CorR if procedural risk is acceptable with no contraindications. NVM on SPECT or Echo, if warranted can be further distinguished by CMR/PET^31^, ^37^ into predominant scar versus PVM or VM, which may provide further guidance in CorR decision making. In VM or PVM, with no transmural scar (NVM), revascularization of corresponding anatomical CAD may be considered. In advanced LV remodeling (severely dilated and dysfunctional LV) with VM or PVM, it may take months to years from CorR, to appreciate the improved cardiovascular outcomes including LV functional recovery.^34^ In an adversely remodeled LV with a large scar burden, it is unlikely that CorR would improve outcomes. In addition, CorR procedural risk might outweigh the benefits.^31^

Further research has been ongoing to understand and precisely characterize myocardial ischemia, fibrosis, and LV remodeling and their interaction with a variety of therapeutic strategies in predicting the outcomes. Novel treatment approaches such as stem cell therapies for ICM are under investigation.

## CONFLICT OF INTERESTS

No financial or nonfinancial interests are directly or indirectly related to the work submitted for publication.

## Supporting information

Supplementary Table 1 Comparison of Imaging Techniques of Myocardial viability Testing.

## Data Availability

The data that support the findings of this study are openly available and appropriate references provided.
