# Supplementary material for: Myocardial viability assessment and utility in contemporary management of ischemic cardiomyopathy
Source: Clin Cardiol. 2022 Jan 25;45(2):152–61. doi: 10.1002/clc.23779 (PMC8860488; doi:10.1002/clc.23779)
Supplement: Supplementary file 1 — Supplementary Table 1 Comparison of Imaging Techniques of Myocardial viability Testing. [file CLC-45-152-s001.doc]

**Imaging Modality Viability Marker Sensitivity Specificity Advantages Limitations**

| DS Echocardiography | Demonstration of Contractile reserve | Moderate (70- 80%) | High (80-90%) | No radiation No iodinated contrast Quick Routinely available | Higher false-positive rate |
| --- | --- | --- | --- | --- | --- |
| SPECT MPI (^201^Tl ) | Normal perfusion (>50% of a normal segment) on redistribution imaging | High (80-90%) | Low to moderate (59%) | Traditionally accepted modality | Limited sensitivity Radiation risk Time consuming |
| SPECT MPI (Tc-99m) | Normal perfusion (>50% of a normal segment) | Moderate to high (80-90%) | Moderate (60-70%) | Quicker than ^201^Tl redistribution imaging, myocardial ischemia and LV function assessed simultaneously | Unable to distinguish NVM from PVM Radiation risk |
| PET (^82^Rb ^18^F-FDG) | Perfusion defect with intact Metabolism | High (>90%) | Moderate (60-70%) | Superior diagnostic accuracy to SPECT imaging Less radiation  Less time consuming | Not routinely available; subendocardial infarcts may be missed |
| CMR (DS) | Presence of CR | Moderate to high (80%) | High (90%) | Accurately images myocardial scar Superior spatial resolution differentiates subendocardial from transmural infarct No radiation | Limited outcome data |
| CMR (LGE) | Absence of LGE, <50% transmural extent of LGE | Moderate - high (80-85%) | Moderate (60-70%) | Superior spatial resolution Less radiation | Transmural size and extent of LGE vs viable, subendocardial non LGE myocardium in conjunction with CR with DS should be taken into account |

^201^Tl MPI SPECT: Thallium myocardial perfusion imaging Single photon emission computed tomography; ^82^Rb ^18^F-FDG PET: Rubidium, Flouride Flouodeoxy glucose Positron Emission Tomography; DS: Dobutamine Stress; LGE: Late gadolinium enhancement; CMR: Cardiac magnetic resonance; CR: Contractile reserve
